# Supplementary figures and images for: Integrated multi-omics approach revealed TTNtv c.13254T>G causing dilated cardiomyopathy in mice
Source: PLoS One. 2024 Oct 4;19(10):e0311670. doi: 10.1371/journal.pone.0311670 (PMC11452030; doi:10.1371/journal.pone.0311670)

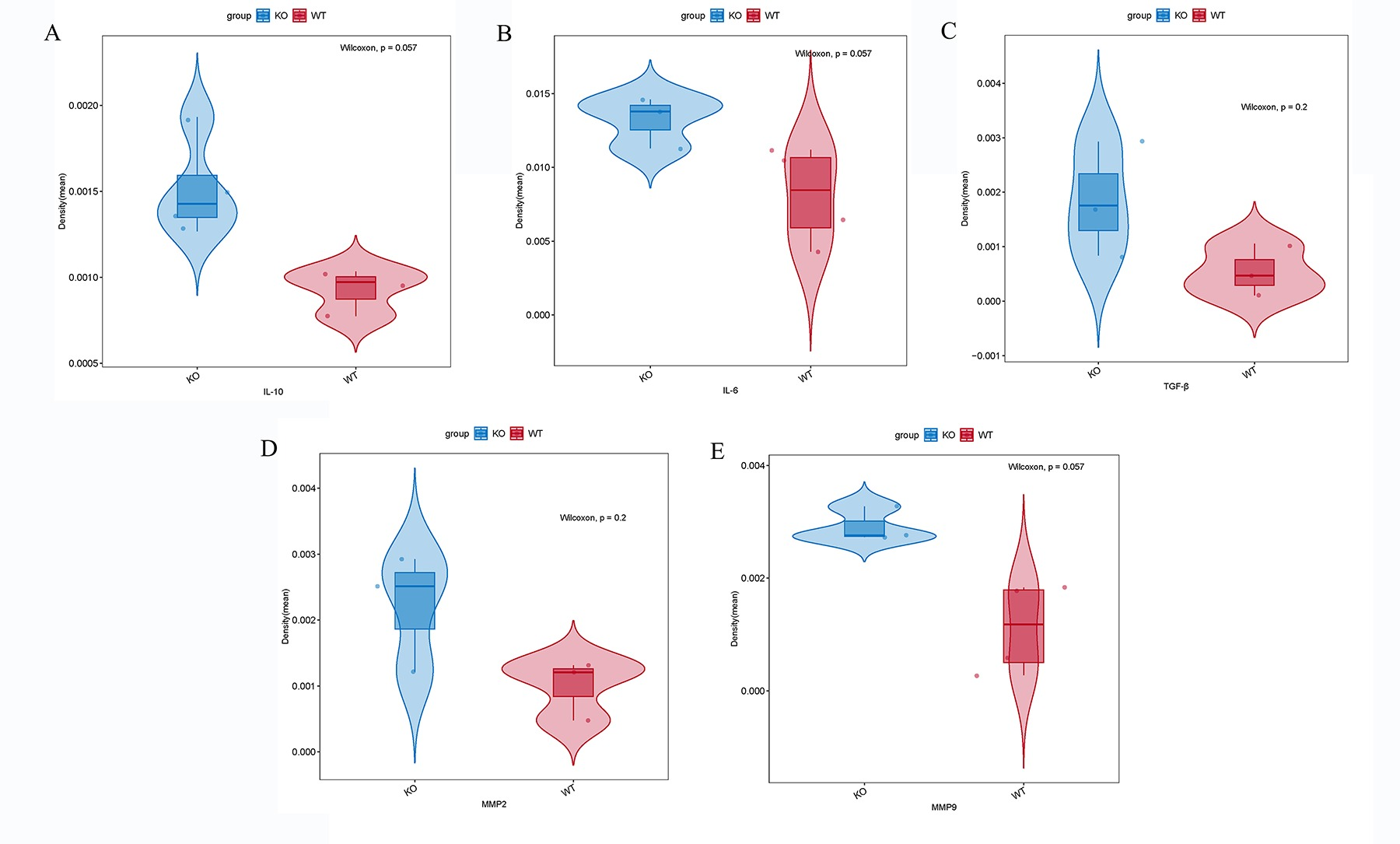

Supplement: S1 Fig — (TIF) [file pone.0311670.s001.tif]

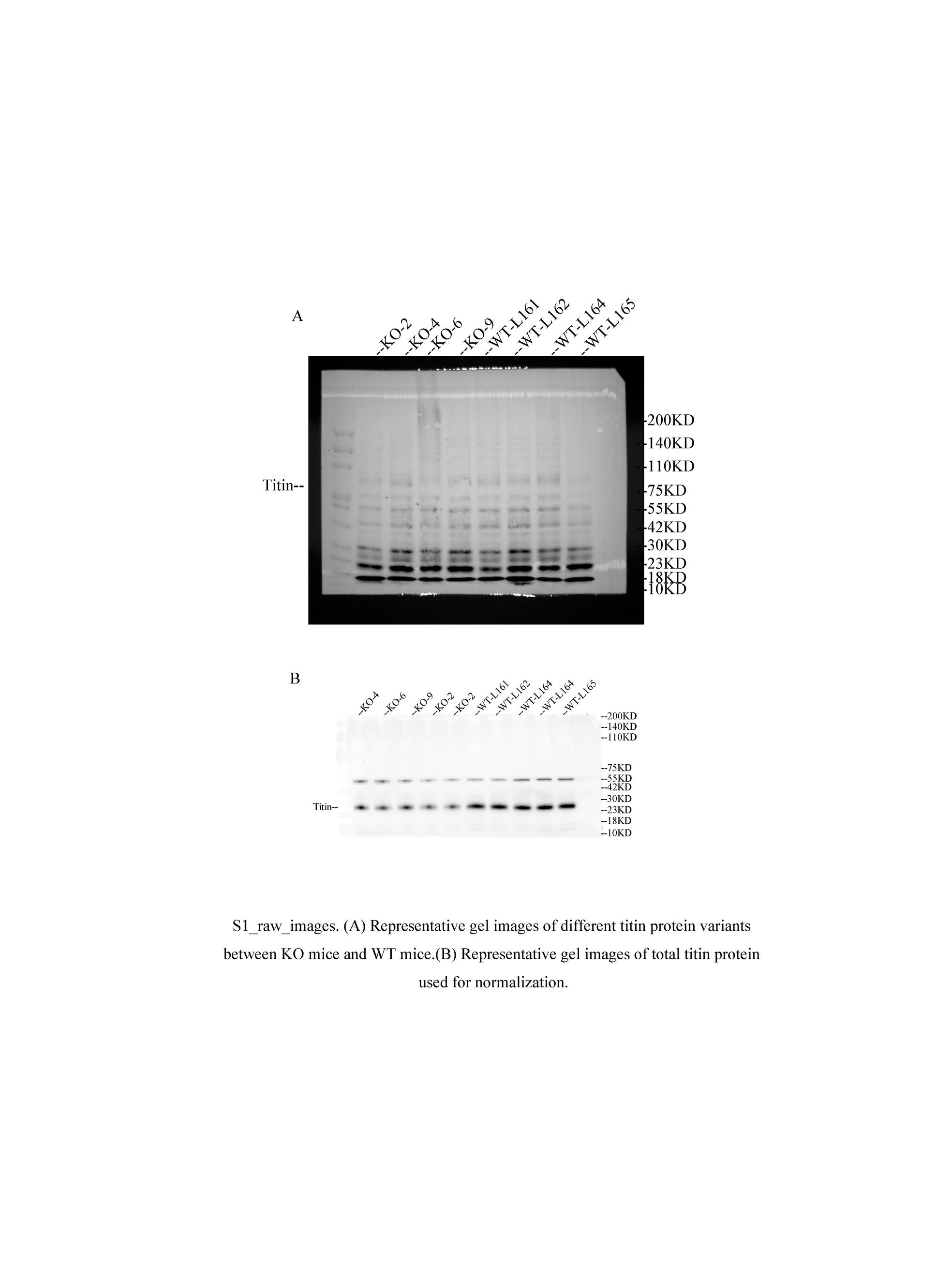

Supplement: S1 Raw image — (A) Representative gel images of different titin protein variants between KO mice and WT mice. (B) Representative gel images of total titin protein used for normalization. (TIF) [file pone.0311670.s002.tif]
