# Supplementary material for: Integrated multi-omics approach revealed TTNtv c.13254T>G causing dilated cardiomyopathy in mice
Source: PLoS One. 2024 Oct 4;19(10):e0311670. doi: 10.1371/journal.pone.0311670 (PMC11452030; doi:10.1371/journal.pone.0311670)
Supplement: Attachment — Submitted filename: Response to Reviewers.docx [file pone.0311670.s003.docx]

Dear editors and reviewers:

Thank you for your letter and for the reviewers’ comments concerning our manuscript entitle “*Integrated multi-omics approach revealed TTNtv c.13254T>G causing dilated cardiomyopathy in mice*” (PONE-D-23-40669). Those comments are all valuable and very helpful for revising and improving our paper, as well as the important guiding significance to our researches. We online submitted a rebuttal letter, a marked-up copy of the manuscript that highlights changes, an unmarked version of the revised paper, a list of responses (with one point by point response), a new cover letter, two reprepared figures (Fig1 and Fig3) processed by PACE, western blot figure of total Titin protein (S2 Fig), and a PDF file named “S1_raw_images” containing all original blot and gel results. We have studied comments carefully and have made correction which we hope meet with approval. Revised portion are highlighted in yellow in the paper. The main corrections in the paper and the responds to the reviewer’s comments are as following:

**Responds and rebuttal to the comments of academic editor and reviewer(s):**

**Journal Requirements:**

1. When submitting your revision, we need you to address these additional requirements. Please ensure that your manuscript meets PLOS ONE's style requirements, including those for file naming.

***Response:*** Thanks for your important comments. We have examined our manuscript carefully, and the manuscript meets PLOS ONE's style requirements, including those for file naming.

**2.** To comply with PLOS ONE submissions requirements, in your Methods section, please provide additional information regarding the experiments involving animals and ensure you have included details on (1) methods of sacrifice, (2) methods of anesthesia and/or analgesia, and (3) efforts to alleviate suffering.

***Response:*** Thanks for your important comments. In the Methods section, we have supplemented details on (1) methods of sacrifice, (2) methods of anesthesia and/or analgesia, and (3) efforts to alleviate suffering. The content of methods of sacrifice “Mice were euthanized by cervical dislocation after anesthesia. Mice were subjected to induced anesthesia with 5% isoflurane until completely stationary, the corneal reflex disappeared, and no pain response was observed when the tail was clamped. Then, the anesthetized mice were placed on a wire mesh. The experimenter pulled the tail of the mouse with one hand, pressed the neck of the mouse with tweezers, and pulled the mouse with hands to dislocate the cervical spine” was added (Page 5, line 2-8). The content of methods of anesthesia and/or analgesia “The mice were anesthetized with a mixture of isoflurane and oxygen. After connecting the pipeline, the anesthesia induction mode with an isoflurane concentration of 3 % was set, and the diverter was opened to input the mixture of isoflurane and oxygen into the anesthesia induction box for a 1-minute induced anesthesia. After induced anesthesia, the concentration of isoflurane was adjusted to 1.5 % ~ 2 % to maintain anesthesia” was added (Page 5, line 9-15). The content of efforts to alleviate suffering “Subcutaneous injection of 0.05-0.1mg/kg buprenorphine was carried out to relieve pain when abnormal pain or urgent response occurred in mice, and intraperitoneal injection of diazepam 5 mg/kg was applied for sedation when mice were nervous” was added (Page 5, line 16-19).

**3.** PLOS ONE now requires that authors provide the original uncropped and unadjusted images underlying all blot or gel results reported in a submission’s figures or Supporting Information files. This policy and the journal’s other requirements for blot/gel reporting and figure preparation are described in detail at [https://journals.plos.org/plosone/s/figures#loc-blot-and-gel-reporting-requirements](https://journals.plos.org/plosone/s/figures%23loc-blot-and-gel-reporting-requirements) and [https://journals.plos.org/plosone/s/figures#loc-preparing-figures-from-image-files](https://journals.plos.org/plosone/s/figures%23loc-preparing-figures-from-image-files). When you submit your revised manuscript, please ensure that your figures adhere fully to these guidelines and provide the original underlying images for all blot or gel data reported in your submission. See the following link for instructions on providing the original image data: [https://journals.plos.org/plosone/s/figures#loc-original-images-for-blots-and-gels](https://journals.plos.org/plosone/s/figures%23loc-original-images-for-blots-and-gels).

***Response:*** Thanks for your important comments. We have created a single PDF file named ‘S1_raw_images’ that contains all the original blot and gel images contained in the manuscript’s main figures. The original images have been compiled and annotated using Photoshop and then exported as a tiff file with LZW compression. The PDF file has been uploaded as a Supporting Information file.

**4.** Thank you for stating the following financial disclosure: [This study is supported by Hubei Natural Science Foundation Youth Program (2022CFB841) and Scientific Research Program of Wuhan Municipal Health Commission (WX20D15). Dr Wenqing Sun received these fundings.]. Please state what role the funders took in the study. If the funders had no role, please state: ""The funders had no role in study design, data collection and analysis, decision to publish, or preparation of the manuscript."" If this statement is not correct you must amend it as needed. Please include this amended Role of Funder statement in your cover letter; we will change the online submission form on your behalf.

***Response:*** Thanks for your important comments. Apologies for mistaking the funding. We have replaced fundings “Hubei Natural Science Foundation Youth Program (2022CFB841) and Scientific Research Program of Wuhan Municipal Health Commission (WX20D15)” by “Special Project of Hubei Provincial Health Commission (WJ2021M033)”. Dr Dan Yu received the funding. The role of the funder Dan Yu has been stated in the author contribution section of the study. Dan Yu participated in study design, conducted formal analysis and methodology. In addition, she wrote original draft and reviewed & edited the manuscript. We have provided an amended statement that declares all the funding or sources of support (whether external or internal to your organization) received during this study. We have included an amended Funding Statement within the cover letter.

**5.** Thank you for stating the following in the Acknowledgments Section of your manuscript: [We thank Hubei Natural Science Foundation Youth Program (2022CFB841) and Scientific Research Program of Wuhan Municipal Health Commission (WX20D15) for funding this study. We are grateful for the technical support provided by BestNovo (Beijing) Medical Laboratory.] We note that you have provided funding information that is not currently declared in your Funding Statement. However, funding information should not appear in the Acknowledgments section or other areas of your manuscript. We will only publish funding information present in the Funding Statement section of the online submission form. Please remove any funding-related text from the manuscript and let us know how you would like to update your Funding Statement. Currently, your Funding Statement reads as follows: [This study is supported by Hubei Natural Science Foundation Youth Program (2022CFB841) and Scientific Research Program of Wuhan Municipal Health Commission (WX20D15). Dr Wenqing Sun received these fundings.]. Please include your amended statements within your cover letter; we will change the online submission form on your behalf.

***Response:*** Thanks for your important comments. We have deleted the funding information from Acknowledgements Section of the manuscript.

6. Thank you for uploading your study's underlying data set. Unfortunately, the repository you have noted in your Data Availability statement does not qualify as an acceptable data repository according to PLOS's standards. At this time, please upload the minimal data set necessary to replicate your study's findings to a stable, public repository (such as figshare or Dryad) and provide us with the relevant URLs, DOIs, or accession numbers that may be used to access these data. For a list of recommended repositories and additional information on PLOS standards for data deposition, please see <https://journals.plos.org/plosone/s/recommended-repositories>.

***Response:*** Thanks for your important comments. We have uploaded the data in the repository DRYAD database with DOI <https://doi.org/10.5061/dryad.tmpg4f563> according to PLOS’s standards.

7. Your ethics statement should only appear in the Methods section of your manuscript. If your ethics statement is written in any section besides the Methods, please delete it from any other section.

***Response:*** Thanks for your important comments. We have deleted the ethics statement written in any section besides the methods.

8. We note that Figure 1 in your submission contain copyrighted images. All PLOS content is published under the Creative Commons Attribution License (CC BY 4.0), which means that the manuscript, images, and Supporting Information files will be freely available online, and any third party is permitted to access, download, copy, distribute, and use these materials in any way, even commercially, with proper attribution. For more information, see our copyright guidelines: <http://journals.plos.org/plosone/s/licenses-and-copyright>. We require you to either (1) present written permission from the copyright holder to publish these figures specifically under the CC BY 4.0 license, or (2) remove the figures from your submission: a. You may seek permission from the original copyright holder of Figure 1 to publish the content specifically under the CC BY 4.0 license. We recommend that you contact the original copyright holder with the Content Permission Form (http://journals.plos.org/plosone/s/file?id=7c09/content-permission-form.pdf) and the following text:“I request permission for the open-access journal PLOS ONE to publish XXX under the Creative Commons Attribution License (CCAL) CC BY 4.0 (http://creativecommons.org/licenses/by/4.0/). Please be aware that this license allows unrestricted use and distribution, even commercially, by third parties. Please reply and provide explicit written permission to publish XXX under a CC BY license and complete the attached form.” Please upload the completed Content Permission Form or other proof of granted permissions as an ""Other"" file with your submission. In the figure caption of the copyrighted figure, please include the following text: “Reprinted from [ref] under a CC BY license, with permission from [name of publisher], original copyright [original copyright year].” b. If you are unable to obtain permission from the original copyright holder to publish these figures under the CC BY 4.0 license or if the copyright holder’s requirements are incompatible with the CC BY 4.0 license, please either i) remove the figure or ii) supply a replacement figure that complies with the CC BY 4.0 license. Please check copyright information on all replacement figures and update the figure caption with source information. If applicable, please specify in the figure caption text when a figure is similar but not identical to the original image and is therefore for illustrative purposes only.

***Response:*** Thanks for your important comments. Although we do not know why there are copyrighted images in Figure 1, we guarantee that we own the original copyright of all images in Figure 1. This problem might be due to the unknown parameter error that occurred in the use of drawing software during the preparation of Fig 1. Now, Fig 1 has been reprepared and uploaded. We apologize for the error.

**Comments to the Author:**

1. Is the manuscript technically sound, and do the data support the conclusions? The manuscript must describe a technically sound piece of scientific research with data that supports the conclusions. Experiments must have been conducted rigorously, with appropriate controls, replication, and sample sizes. The conclusions must be drawn appropriately based on the data presented.

Reviewer #1: No

Reviewer #2: Yes

***Response:*** Thanks for your important comments. Thank you very much.

2. Has the statistical analysis been performed appropriately and rigorously?

Reviewer #1: I Don't Know

Reviewer #2: Yes

***Response:*** Thanks for your important comments. We have examined our statistical analysis program of the manuscript carefully, and make sure that the statistical analysis appropriately and rigorously.

3. Have the authors made all data underlying the findings in their manuscript fully available? The [PLOS Data policy](http://www.plosone.org/static/policies.action#sharing) requires authors to make all data underlying the findings described in their manuscript fully available without restriction, with rare exception (please refer to the Data Availability Statement in the manuscript PDF file). The data should be provided as part of the manuscript or its supporting information, or deposited to a public repository. For example, in addition to summary statistics, the data points behind means, medians and variance measures should be available. If there are restrictions on publicly sharing data—e.g. participant privacy or use of data from a third party—those must be specified.

Reviewer #1: No

Reviewer #2: Yes

***Response:*** Thanks for your important comments. We have uploaded all data as PLOS Data policy required. Thank you very much.

4. Is the manuscript presented in an intelligible fashion and written in standard English? PLOS ONE does not copyedit accepted manuscripts, so the language in submitted articles must be clear, correct, and unambiguous. Any typographical or grammatical errors should be corrected at revision, so please note any specific errors here.

Reviewer #1: No

Reviewer #2: Yes

***Response:*** Thanks for your important comments. We have made every effort to polish the language. Thank you very much.

**5. Review Comments to the Author:**

**Reviewer #1**

Reviewer #1: Authors performed extensive experimental search to analyze functional effect but did not present and prioritize their data properly. Many important points should be addressed to improve the quality of this manuscript.

***Response:*** Thanks for your important comments. Thank you very much.

**Comments to review：**

1. There is neither clinical description of patients nor phenotype of the model mice. Authors statement “In previous study, we reported two probands diagnosed with DCM carrying a novel TTNtv c.13254T>G (p.Tyr4418Ter)…” does not supported by any data. No citation to find this previous study, no published clinical case in PubMed with this mutation under given authorships, no any minimal clinical data about patients (at least age, gender, instrumental investigation, time of follow-up, any epigenetic factors of DCM etc. No description of the phenotype in mice, time of DCM detection, heart failure progression, comparability of the human and mourine cardiac involvement.

***Response:*** Thanks for your important comments. We are very sorry for forgetting to cite our previous study “*The TTN p. Tyr4418Ter mutation causes cardiomyopathy in human and mice*”. Now the previous study has been cited in the introduction section (page 3, line 24) and discussion section (Page 17, line 5). In the previous study, the clinical data about two probands was described in detail in the case reports section. The construction process of KO mice, validation by sanger sequencing, serological detection time, echocardiography detection time, histological staining time were also described in the materials and methods section.

2. Genetic data are strongly underrepresented even zygosity of the TTNtv rare variant in human and mice did not mention. The volume of genetic testing is required. Are there other candidate findings? The correct description of the variant of interest is absent (genomic coordinate with the reference genome version (hg19/hg38), correct MANE or MANE Clinical transcript number or RefSeq used for cDNA description. Considering multiple transcripts for TTN gene this information in crucial. Authors mentioned once 363-exons transcript, and it might me a virtual Meta-transcript (ENST00000589042/hg19). If so than exon 45 in Meta would not present in N2B and N2BA (principal long cardiac isoforms) and expresses only in novex-1 isoform (minor short cardiac isoform), and PSI (percentage spliced in) for this exon in myocardium as small as 1%. It raises the question about ACMG (2015) criteria chosen for this variant to get Class III of pathogenicity. How it goes in mice?

***Response:*** Thanks for your important comments. Human TTN gene is located on chromosome 2. The TTNtv c.13254T>G found out in our study was on the transcript variant novex-3 NM_133379.5 and its location on the genome was chr2: 179613873. The TTNtv c.13254T>G was on exon 46 in human and TTNtv p.Y4370X equal to TTNtv c.13254T>G was on exon 45 in mice (transcript XM_036160543.1-Ttn-202-ENSMUST00000099980.10). The human transcript mRNA NM_133379.5 has 5604 amino acids, of which TTNtvc.13254T>G resulted in an advance stop codon at the 4418 amino acid position, resulting in protein truncation. As we mentioned in the previous study, the downstream loci c.10120A>T (p.Lys3374Ter), c.5383A>T (p.Lys1795Ter), and c.5047C>T (P.RG1683ter) of TTN gene have all been reported as pathogenic mutations of dilated cardiomyopathy (NM_001267550.2), so the TTNtv c.13254T>G can lead to TTN protein truncation changes, and then lead to dilated cardiomyopathy. Similarly, the mouse transcript XM_036160543.1 has 5508 amino acids, while TTNtv p.Y4370X resulted in the generation of a truncated protein with 4370 amino acids. Our previous study proved that two-month-old TTN KO mice had myocardial injury detected by serological detection and echocardiography. Histology results showed that cardiac fibrosis level and positive rate of cardiac mast cell of TTN+/- mice were obviously increased compared with WT mice, and the heart weight of TTN+/- mice had an increasing trend, manifesting the abnormal trend of TTN+/- mice’s myocardial structure.

3. Does this variant undergo NMD elimination or escape NMD? This should be checked. What happens with total mRNA in human and mice? Human exon 45 (if correctly determined) in symmetric and the loss of exon 45 does not change the ORF in novex-1 transcript.

***Response:*** Thanks for your important comments. Usually, when the distance between PTC (premature termination codon) and the last EEJ (exon-exon junction) is greater than or equal to 50-55 nucleotides, NMD can be triggered, known as the 50 boundary rule of the NMD pathway. However, the TTNtv c.13254T>G was located on the last exon 46 of *TTN* in human, which does not comply with this rule. The TTNtv c.13254T>G cannot trigger NMD pathway. Sorry that total mRNA in the two probands with DCM was not detected. The results of transcriptome sequencing of cardiac tissue samples from the two mouse groups revealed significant differences in pathways associated with immune regulation and inflammatory responses. According to the requirement of Reviewer #2, we quantified total titin protein of cardiac tissues from KO and WT mouse groups by western blot, indicating a significant decrease on total titin protein of KO mice cardiac tissues. As we mentioned in the response to the question 2, the TTNtv c.13254T>G was located on human exon 46 and TTNtv p.Y4370X equal to TTNtv c.13254T>G was located on mouse exon 45. Both mutations did lead to the generation of truncated proteins.

4. Detailed description of the DNA findings, RNA spectrum and quantity are important for understanding what’s going on in myocardium with this rare variant.

***Response:*** Thanks for your important comments. The DNA findings were described in detail in our previous study, where we reported clinical data of two probands with DCM and the finding of *TTNtv* c.13254T>G by whole exon sequencing. Based on DNA findings, we designed and constructed heterozygous KO mice carrying TTNtv p.Y4370X, equal to *TTNtv* c.13254T>G, validated by Sanger sequencing. Regarding RNA spectrum, differentially expressed genes mostly enriched in biological process, molecular function and pathways primarily related to development and regulation of cardiac muscle contraction, actin and actin filament binding and organization, regulation of actin cytoskeleton, DCM, HCM, manifesting that truncation of titin might affect the biological process and molecular function of these DEGs and led to myocardial injury.

5. Figures with histological findings are accompanied with nice statistics plots. But it is completely unclear what parameter authors try to compare.

***Response:*** Thanks for your important comments. The parameters we tried to compare were inflammatory factors IL-6, IL-10, TNF-α, TGF-β, MMP2, MMP9 and cTNT to validate whether cardiac structure alteration happened in KO mice, which has been added in the Immunohistochemistry section of materials and methods (Page 6, line 1-4).

6. Discussion will largely depend on improved phenotypic and genetic description.

***Response:*** Thanks for your important comments. We have supplemented phenotypic and genetic description in the first paragraph of Discussion section. In previous study, we found a novel TTNtv c.13254T>G in two probands with DCM but classified as versus uncertain significance (VUS) by American College of Medical Genetics and Genomics (ACMG). Both probands are females, aged 62 and 52 respectively. The results of echocardiography and cardiac magnetic resonance imaging both showed that proband 1 had left heart enlargement, decreased amplitude of ventricular septal and left ventricular wall movement, and decreased left ventricular systolic function. Myocardial delayed imaging showed the formation of myocardial fibrosis in the interventricular septum, left anterior wall, and inferior wall of the myocardium. The echocardiography results of proband 2 showed enlargement of the left atrium and left ventricle, and a general decrease in the amplitude of ventricular septal and left ventricular wall movement. The cardiac magnetic resonance imaging results showed left heart enlargement, segmental motion abnormalities in the left ventricular wall, severe reduction in left ventricular systolic function, and moderate reduction in right ventricular systolic function, presenting as dilated cardiomyopathy with myocardial infarction. Whole exon sequencing revealed that two probands carried a TTNtvc.13254T>G (p.Tyr4418Ter) heterozygous mutation located in transcript NM_133379.5 (chr2:179613873). The mother of proband 1 suffered from heart disease and died of repeated heart failure. Proband 1 had 6 siblings and her sister suffered from heart disease (cardiac enlargement, details unknown). Due to the fact that proband 1 did not provide samples and clinical data of family members, it was not possible to verify whether her family members carried the mutation. The father of proband 2 died for unknown reasons at 40 years old, and the daughter carried the same heterozygous mutation (Page 16, line 1-23).

**Reviewer #2**

Reviewer #2: The present study revealed the association of TTNtv c. 13254T>G in dilated cardiomyopathy in murine model. The manuscript is well written in standard English and statistical analysis is properly done.

***Response:*** Thanks for your important comments. Thank you very much.

**Comments to review：**

1. Western blotting results quantification and graph (Figure 3) for different titin protein variants are not convincing and required to be re-quantified. Although the Gapdh is shown to stable between KO and WT samples, it would be more appropriated to normalize the band intensities with the total protein. Additionally, a full gel picture with molecular marker would be very helpful (either in supplement or in main manuscript).

***Response:*** Thanks for your important comments. We have re-performed Wilcoxon test with normalized gray value of different titin protein bands and the Fig3 was reprepared. We also conducted Western blot to quantify the total titin protein between KO and WT mice. The full gel picture with molecular marker was provided as a supplementary file named “S2 figure”. Results showed that total Titin protein in KO mice was significantly decreased compared to WT mice.

2. Figure legends should be more informative and required to be rewritten.

***Response:*** Thanks for your important comments. We have complemented more information to figure legends.

3. Manuscript has some syntaxes and easily recoverable errors.

***Response:*** Thanks for your important comments. We have tried our best to correct these syntaxes and errors.

4. Method section required some more Information regarding reagents used (supplier, name of kits, catalogue no. etc).

***Response:*** Thanks for your important comments. The information regarding reagents used like supplier, name of kits, catalogue no. etc have been added.

5. At few places the selection of scientific words will be more appropriate (For example: instead of lifted ..use upregulated/ increased etc)

***Response:*** Thanks for your important comments. We have replaced these words with more appropriate words.

Special thanks to you for your good comments.

We tried our best to improve the manuscript and made some changes in the manuscript. According to the comments of reviewers, we have revised the manuscript and list specific changes here point by point. The changes have been highlighted in yellow in revised paper. The table line number of specific changes is based on the new revised manuscript.

We appreciate for Editors/Reviewers’ warm work earnestly, and hope that the correction will meet with approval.

Once again, thank you very much for your comments and suggestions.

***The list of specific changes***

1. **Page 1, line 8**

The authors and the order of authors were adjusted. “Wenqiang Sun^1^, Kaisheng Lai^2^, Xiaohui Liu^3^, Laichun Song^1^, Liang Tao^1^, Hui Jiang^2^, Hongyan Xiao^1*^” was replaced by “Dan Yu^1^, Liang Tao^1^, Laichun Song^1^, Kaisheng Lai^2^, Hui Jiang^2^, Zhe Liu^2^, Hongyan Xiao^1*^”.

2. **Page1, line 16**

The affiliation “^3^ Department of Clinical Laboratory, Wuhan Asia Heart Hospital Affiliated with the Wuhan University of Science and Technology, Wuhan, Hubei, 430022, P.R. China.” was removed.

3. **Page 2, line1**

The format of level 1 heading ”Abstract” was adjusted as required.

4. **Page 2, line 9**

“and” was added.

5. **Page 2, line 10**

“of” was replaced by “on”.

6. **Page 2, line 11**

“observe” was replaced by “observe”.

7. **Page 2, line 16**

“a” was inserted between “caused” and “decrease”.

8. **Page 2, line 18-19**

“Summary” was replaced by “In summary”.

9. **Page 2, line 19**

“is contributed” was replaced by “contributed”.

10. **Page 3, line 3**

“2 / 3” was replaced by “2/3”.

11. **Page 3, line 4**

“1 / 3” was replaced by “1/3”.

12. **Page 3, line 19-20**

“on exon 46 of TTN (the transcript was NM_133379.5)” was added.

13. **Page 3, line 24**

“[11]” was inserted.

14. **Page 4, line 15-19**

Sentence “Sequence indicates gRNA cut site on exon 45 of the mouse TTN gene was selected as the target site.” was replaced by “Sequence (GATGATGTTGGATATCATGGACCGGACTGGGGAAATATGAAAGGACATTCTCAAAGTGAT(TAT)GTGCTAAAT(ATC)CACTC//CAAGAGAACCTCTAACACAGTTCAGGACGTGGAAGACTCACCTGTCCCTACCTAT, “//” indicates gRNA cut site) on exon 45 of the mouse TTN gene was selected as the target site.”.

15. **Page 5, line 2**

“Methods of sacrifice” was added.

16. **Page 5, line 3-8**

“Mice were euthanized by cervical dislocation after anesthesia. Mice were subjected to induced anesthesia with 5% isoflurane until completely stationary, the corneal reflex disappeared, and no pain response was observed when the tail was clamped. Then, the anesthetized mice were placed on a wire mesh. The experimenter pulled the tail of the mouse with one hand, pressed the neck of the mouse with tweezers, and pulled the mouse with hands to dislocate the cervical spine” was added.

17. **Page 5, line 9**

“Methods of anesthesia and/or analgesia” was added.

18. **Page 5, line 10-15**

“The mice were anesthetized with a mixture of isoflurane and oxygen. After connecting the pipeline, the anesthesia induction mode with an isoflurane concentration of 3 % was set, and the diverter was opened to input the mixture of isoflurane and oxygen into the anesthesia induction box for a 1-minute induced anesthesia. After induced anesthesia, the concentration of isoflurane was adjusted to 1.5 % ~ 2 % to maintain anesthesia” was added.

19. **Page 5, line 16**

“Efforts to alleviate suffering” was added.

20. **Page 5, line 17-19**

“Subcutaneous injection of 0.05-0.1mg/kg buprenorphine was carried out to relieve pain when abnormal pain or urgent response occurred in mice, and intraperitoneal injection of diazepam 5 mg/kg was applied for sedation when mice were nervous” was added.

21. **Page 5, line 23-24**

“(Servicebio, Wuhan, China)” was replaced by “(G1102, Wuhan Servicebio Technology Co., Ltd., China)”. “(HT7800/HT7700, Hitachi, Japan)” was added.

22. **Page 6, line 10-13**

The sentence “Immunohistochemistry was applied to detect the difference of inflammatory factors IL-6, IL-10, TNF-α, TGF-β, MMP2, MMP9 and cTNT of cardiac tissue between KO mice and WT mice to validate whether cardiac structure alteration happened in KO mice” was added.

23. **Page 6, line 13-14**

“(10009218, Sinopharm Chemical Reagent Co., Ltd., China)” was added.

24. **Page 6, line 18-19**

“(69019361, Wuhan Servicebio Technology Co., Ltd., China)” was added.

25. **Page 6, line 20-21**

“(DD13, Talentbiomedical, China)” was added.

26. **Page 6, line 24**

“(G1212-200T, Wuhan Servicebio Technology Co., Ltd., China)” was added.

27. **Page 6, line 25**

“(DS-Fi3, Nikon, Japan)” was added.

28. **Page 7, line 2**

“(H9627, Sigma, Germany)” was added.

29. **Page 7, line 5-6**

“(10023418, Sinopharm Chemical Reagent Co., Ltd., China)” was added.

30. **Page 7, line 7-8**

“(10004160, Sinopharm Chemical Reagent Co., Ltd., China)” was added.

31. **Page 7, line 10**

“or collect images” after “under a microscope” was deleted.

32. **Page 7, line 15**

“(20-188, Merck, Germany)” was added.

33. **Page 7, line 16**

“(P0015, CWBIO, China)” was added.

34. **Page 7, line 18**

“(P0012AC, CWBIO, China)” was added.

35. **Page 7, line 22-23**

“(0497, Amersco, USA)” was added.

36. **Page 7, line 23**

“(0167, Amersco, USA)” was added.

37. **Page 7, line 23-24**

“(10014108, Sinopharm Chemical Reagent Co., Ltd., China) transfer buffer” was added.

38. **Page 7, line 25, and page 8, line 1**

“skimmed milk powder (TBST)” was replaced by “DifcoTM Skim Milk (232100, BD, USA)”.

39. **Page 8, line 2**

“(72015460, Sinopharm Chemical Reagent Co., Ltd., China)” was added.

40. **Page 8, line 3-4**

“(titin, Abcam, UK; GAPDH, GOODHERE BIOTECH, Hangzhou, China)” was replaced by “(titin, ab284860, Abcam, UK; GAPDH, AB-P-R 001, GOODHERE BIOTECH, Hangzhou, China)”.

41. **Page 8, line 4-5**

“BSA” was replaced by “bovine serum albumin (BSA) (C134730100, Sinopharm Chemical Reagent Co., Ltd., China)”.

42. **Page 8, line 7**

“(WD-9405F, BEIJING LIUYI BIOTECHNOLOGY CO., LTD., China)” was added.

43. **Page 8, line 9-10**

“(Beyotime Biotechnology, Beijing, China) ” was replaced by “(A0208, Beyotime Biotechnology, Beijing, China)”

44. **Page 8, line 10**

“skim milk powder” was replaced by “skim milk”

45. **Page 8, line 15**

“a chemiluminescence camera” was replaced by “the multifunctional microplate reader”.

46. **Page 8, line 15-16**

“(EnSpire, PerkinElmer, USA)” was added.

47. **Page 8, line 16**

“(32106, Thermo Scientific, USA)” was added.

48. **Page 8, line 19**

“12183018A,” was added.

49. **Page 8, line 20-22**

“(Agilent Technologies, USA)” was replaced by “(Agilent Technologies Inc, USA) using RNA 6000 Nano kit 5067-1511 (Agilent Technologies Inc, USA)”

50. **Page 8, line 24**

“NEBNext® Ultra™ RNA Library Prep Kit” was replaced by “NEBNext Ultra II RNA Library Prep Kit”.

51. **Page 8, line 25**

“(Illumina, USA)” was replaced by “(E7770L, New England Biolabs Inc, USA)”.

52. **Page 9, line 6-7**

“(Agilent Technologies Inc, USA) using Agilent High Sensitivity DNA Kit (5067-4626, Agilent Technologies Inc, USA)” was added.

53. **Page 9, line 15-16**

“the Illumina sequencing platform” was replaced by “Illumina NovaSeq 6000 platform (Shanghai Personal Biotechnology Cp. Ltd, China) in PE150 mode”.

54. **Page 9, line 19**

“(M109-5G, Amresco, USA)” was added.

55. **Page 9, line 20-21**

“(M216-30G, Amresco, USA)” was added.

56. **Page 9, line 22-23**

“(A6141-500G, Sigma-Aldrich, Germany)” was added.

57. **Page 9, line 23**

“(V5280/100ug, Promega, USA)” was added.

58. **Page 9, line 25**

“(T79708, Sigma-Aldrich, Germany)” was added.

59. **Page 10, line 2**

“186003581,” was added. “(34851 MSDS, J.T.Baker, North America)” was added.

60. **Page 10, line 5-6**

“Wash off impurities, and finally use 70 % acetonitrile to elute, collect the flow through liquid, freeze-dried.” was replaced by “Impurities were washed off, and finally 70 % acetonitrile was used to elute. The flow-through was collected and freeze-dried.”.

61. **Page 10, line 6-7**

“from TMT10plex™ Isobaric Label Reagent Set (90111, Thermo Fisher Scientific, USA)” was added.

62. **Page 10, line 9**

“(013-23355, Wako Pure Chemical Industries Ltd, Japan)” was added.

63. **Page 10, line 10**

“Vortex” was replaced by “vortex”.

64. **Page 10, line 11-17**

“The mixed labeled samples were dissolved with 100 μ mobile phase A followed by 14000g centrifuge for 20 minutes. Then the supernatant was taken, and high-performance liquid phase was used for classification treatment. Nanoflow LC-MS/MS analysis of tryptic peptides was conducted on a quadrupole Orbitrap mass spectrometer (Q Exactive HF-X, Thermo Fisher Scientific, Bremen, Germany) coupled to an EASY nLC 1200 ultra-high pressure system (Thermo Fisher Scientific) via a nano-electrospray ion source.” was added.

65. **Page 10, line 19**

“m / z” was replaced by “m/z”.

66. **Page 10, line 19-20**

“The maximum injection time of C-trap is 50 ms ;” was replaced by “The resolution of the primary mass spectrometry was set to 120000 (200 m/z), with an AGC of 4e5, and the maximum injection time of C-trap is 50 ms.”.

67. **Page 10, line 21**

“the ' Top Speed '” was replaced by “The 'Top Speed'”.

68. **Page 10, line 21-23**

“The resolution of the secondary mass spectrometry was set to 30000 (200 m / z), AGC was 5e^4^, the maximum injection time was 54 ms,” was replaced by “The resolution of the secondary mass spectrometry was set to 30000 (200 m/z) with the AGC of 5e^4^ and a maximum injection time of 54 ms.”.

69. **Page 10, line 23**

“and the” was replaced by “The”.

70. **Page 11, line 13**

The format of level 2 heading ”Statistics” was adjusted as required.

71. **Page 11, line 16**

“were” was replaced “was”.

72. **Page 12, line 10**

“Fig. 1A” was replaced by “Fig 1A”.

73. **Page 13, line 6-10**

“The myocardial tissue of KO mice # 2, # 4, and # 6 was most severely damaged, with obvious cytoplasmic edema. More myofibrillar fibers dissolved and disappeared. A few myocardial tissues exhibited sparse myofilaments and symmetrical sarcomeres. Mitochondria were increased in number and aggregated. The myocardial damage of KO mouse # 9 was milder.” was added.

74. **Page 13, line 11-19**

“WT mice L161, L162, and L165 showed the least damage, with uniform cytoplasmic density and tightly arranged myofibrils. Part of the myocardial tissue had a small number of broken myofibrils and sparse muscle filaments. Most sarcomere are symmetrically distributed. The number of mitochondria was acceptable. The myocardial tissue damage of WT mouse L164 was second to that of KO mice # 2, # 4, and # 6, with slight cytoplasmic edema, uneven thickness of myofibrils, dense muscle filament structure, and symmetrical distribution of sarcomeres. The number of mitochondria had no significant increase, and their distribution was relatively uniform.” was added.

75. **Page 13, line 25**

“Fig. 2” was replaced by “Fig 2”.

76. **Page 14, line 6**

“lifted” was replaced by “upregulated”.

77. **Page 14, line 10**

“and 3B” was removed.

78. **Page 14, line 13**

“(Figs 3C-3H)” was replaced by “(Figs 3B-3G)”.

79. **Page 14, line 13-14**

“Additionally, the total TTN protein of KO mice was significantly lower than that of WT mice (S2 Fig).” was added.

80. **Page 14, line 16**

“(B)~(H)” was replaced by “(B)~(G)”.

81. **Page 15, line 1**

“up-regulated” was replaced by “upregulated”.

82. **Page 15, line 13**

“Fig. 5D” was replaced by “Fig 5D”.

83. **Page 15, line 22**

“cla	ssified” was replaced by “classified”.

84. **Page 16, line 1**

“Fig. 6A” was replaced by “Fig 6A”.

85. **Page 16, line 6**

“Fig. 6B” was replaced by “Fig 6B”.

86. **Page 16, line 10-25, and page 17, line 1-7**

“In previous study, we found a novel TTNtv c.13254T>G in two probands with DCM but classified as versus uncertain significance (VUS) by American College of Medical Genetics and Genomics (ACMG). Both probands are females, aged 62 and 52 respectively. The results of echocardiography and cardiac magnetic resonance imaging both showed that proband 1 had left heart enlargement, decreased amplitude of ventricular septal and left ventricular wall movement, and decreased left ventricular systolic function. Myocardial delayed imaging showed the formation of myocardial fibrosis in the interventricular septum, left anterior wall, and inferior wall of the myocardium. The echocardiography results of proband 2 showed enlargement of the left atrium and left ventricle, and a general decrease in the amplitude of ventricular septal and left ventricular wall movement. The cardiac magnetic resonance imaging results showed left heart enlargement, segmental motion abnormalities in the left ventricular wall, severe reduction in left ventricular systolic function, and moderate reduction in right ventricular systolic function, presenting as dilated cardiomyopathy with myocardial infarction. Whole exon sequencing revealed that two probands carried a TTNtvc.13254T>G (p.Tyr4418Ter) heterozygous mutation located in transcript NM_133379.5 (chr2:179613873). The mother of proband 1 suffered from heart disease and died of repeated heart failure. Proband 1 had 6 siblings and her sister suffered from heart disease (cardiac enlargement, details unknown). Due to the fact that proband 1 did not provide samples and clinical data of family members, it was not possible to verify whether her family members carried the mutation. The father of proband 2 died for unknown reasons at 40 years old, and the daughter carried the same heterozygous mutation.” was added.

87. **Page 17, line 14**

Citation “[11]” was inserted.

88. **Page 17, line 15**

“evidences” was replaced by “evidence”.

89. **Page 17, line 17-18**

“TEM indicated that all of KO mice were observed the damage of cardiac tissue, especially # 2, # 4 and # 6 was the most serious” was replaced by “TEM showed that the damage of cardiac tissue happened in all KO mice, with # 2, # 4 and # 6 being the most severely damaged”.

90. **Page 17, line 21-22**

“However, all of WT mice were observed mild or almost normal cardiac muscle damage” was replaced by “However, it was observed that all WT mice had mild or almost normal myocardial damage”.

91. **Page 18, line 10**

“In” was replaced by “in”.

92. **Page 18, line 11**

“were also have” was replaced by “also had”

93. **Page 18, line 15**

“play” was replaced by “plays”.

94. **Page 19, line 15**

“WT” was replaced by “KO”.

95. **Page 19, line 25**

“lifted” was replaced by “increased”.

96. **Page 20, line 1**

“(IL-1)” was removed.

97. **Page 20, line 13-14**

“Financial disclosure” was removed.

98. **Page 20, line 15-16**

“The ethics statement” was removed.

99. **Page 21, line 1-2**

Sentence “We thank Hubei Natural Science Foundation Youth Program (2022CFB841) and Scientific Research Program of Wuhan Municipal Health Commission (WX20D15) for funding this study “ was removed.

100. **Page 21, line 4-7**

Sentence “The sequencing data has been submitted to NCBI SRA database with the accession number PRJNA1048119 and can be accessed with the URL <http://www.ncbi.nlm.nih.gov/bioproject/1048119>.” was replaced by “The sequencing data has been submitted to DRYAD database with DOI https://doi.org/10.5061/dryad.tmpg4f563 and can be accessed with the URL https://doi.org/10.5061/dryad.tmpg4f563.”

101. **Page 22, line 8-11**

Reference “11.	Sun W, Liu X, Song L, Tao L, Lai K, Jiang H, et al. The TTN p. Tyr4418Ter mutation causes cardiomyopathy in human and mice. PloS one. 2024;19(2):e0296802. Epub 2024/02/21. doi: 10.1371/journal.pone.0296802. PubMed PMID: 38381767; PubMed Central PMCID: PMCPMC10880961.” was added.

102. **Page 23, line 16-19**

“S2 Fig. (A) Quantification results of the total titin of KO mice and WT mice by WB. WB was carried out twice on KO mouse #2 and WT mouse L164 heart tissue samples. (B) Wilcoxon test showed that titin's total protein expression level was profoundly declined in KO mice than in WT mice.” was added.
