# Supplementary material for: Integrated multi-omics approach revealed TTNtv c.13254T>G causing dilated cardiomyopathy in mice
Source: PLoS One. 2024 Oct 4;19(10):e0311670. doi: 10.1371/journal.pone.0311670 (PMC11452030; doi:10.1371/journal.pone.0311670)
Supplement: Attachment — Submitted filename: 01 Response to Reviewers.docx [file pone.0311670.s004.docx]

Dear editors and reviewers:

Thank you for your letter and for the reviewers’ comments concerning our manuscript entitled “*Integrated multi-omics approach revealed TTNtv c.13254T>G causing dilated cardiomyopathy in mice*” (PONE-D-23-40669). Those comments are all valuable and very helpful for revising and improving our paper, as well as the important guiding significance to our researches. We online submitted a rebuttal letter, a marked-up copy of the manuscript that highlights changes, an unmarked version of the revised paper, one reprepared figure (Fig3) processed by PACE. We have studied comments carefully and have made correction which we hope meet with approval. Revised portion are highlighted in yellow in the paper. The main corrections in the paper and the responds to the reviewer’s comments are as following:

**Responds and rebuttal to the comments of academic editor and reviewer(s):**

**Journal Requirements:**

1. Please review your reference list to ensure that it is complete and correct. If you have cited papers that have been retracted, please include the rationale for doing so in the manuscript text, or remove these references and replace them with relevant current references. Any changes to the reference list should be mentioned in the rebuttal letter that accompanies your revised manuscript. If you need to cite a retracted article, indicate the article’s retracted status in the References list and also include a citation and full reference for the retraction notice.

***Response:*** Thanks for your important comments. We have reviewed the reference list carefully. It is complete and correct without any retracted papers cited. No changes have been made to the reference list.

**Comments to the Author:**

1. If the authors have adequately addressed your comments raised in a previous round of review and you feel that this manuscript is now acceptable for publication, you may indicate that here to bypass the “Comments to the Author” section, enter your conflict of interest statement in the “Confidential to Editor” section, and submit your "Accept" recommendation.

Reviewer #2: (No Response)

***Response:*** Thanks for your important comments. Thank you very much.

2. Is the manuscript technically sound, and do the data support the conclusions?

The manuscript must describe a technically sound piece of scientific research with data that supports the conclusions. Experiments must have been conducted rigorously, with appropriate controls, replication, and sample sizes. The conclusions must be drawn appropriately based on the data presented.

Reviewer #2: Partly

***Response:*** Thanks for your important comments. According to your suggestion, we reevaluated WB data using total protein normalization method.

3. Has the statistical analysis been performed appropriately and rigorously?

Reviewer #2: Yes

***Response:*** Thanks for your important comments. Thank you very much.

4. Have the authors made all data underlying the findings in their manuscript fully available?

The PLOS Data policy requires authors to make all data underlying the findings described in their manuscript fully available without restriction, with rare exception (please refer to the Data Availability Statement in the manuscript PDF file). The data should be provided as part of the manuscript or its supporting information, or deposited to a public repository. For example, in addition to summary statistics, the data points behind means, medians and variance measures should be available. If there are restrictions on publicly sharing data—e.g. participant privacy or use of data from a third party—those must be specified.

Reviewer #2: Yes

***Response:*** Thanks for your important comments. Thank you very much.

5. Is the manuscript presented in an intelligible fashion and written in standard English?

PLOS ONE does not copyedit accepted manuscripts, so the language in submitted articles must be clear, correct, and unambiguous. Any typographical or grammatical errors should be corrected at revision, so please note any specific errors here.

Reviewer #2: Yes

***Response:*** Thanks for your important comments. Thank you very much.

**6. Review Comments to the Author:**

Please use the space provided to explain your answers to the questions above. You may also include additional comments for the author, including concerns about dual publication, research ethics, or publication ethics. (Please upload your review as an attachment if it exceeds 20,000 characters)

Reviewer #2: Thank you for some of recommended changes you have made throughout the manuscript; however, the following points need to be addressed.

Major point:

1) Figure 3: Again, if you look carefully, quantification of 10-18, 18-23, 23-30, 30 KD titin proteins with GAPDH is not correct and convincible. They need to be quantify with adequate precision.

***Response:*** Thanks for your important comments. We apologize for not understanding your suggestions correctly for the first time. We’ve re-quantified 10-18, 18-23, 23-30, 30, 42 and 55 KD titin proteins with total protein existing on experimental blot. Figure 3 has been redrawn with the updated normalized data. (Page 14, line 8-19).

2) There is missing information regarding the software or program and method used for this quantification in text.

***Response:*** Thanks for your important comments. The information of the software and method used for WB quantification has been supplemented to the “Statistics” section. “For western blotting, Image J was used to obtain the grayscale value of the target protein band which was further normalized with total protein” was added (Page 11, line 13-14).

3) To enhance the confidence in Western results, it was suggested that to quantify each titin protein isomer with normalization method using total protein existing on experimental blot. However, it was partially done.

***Response:*** Thanks for your important comments. We apologize for not understanding your suggestions correctly for the first time. According to your advice, we’ve normalized 10-18, 18-23, 23-30, 30, 42 and 55 KD titin proteins using total protein existing on experimental blot.

**7.** PLOS authors have the option to publish the peer review history of their article (what does this mean?). If published, this will include your full peer review and any attached files.

If you choose “no”, your identity will remain anonymous but your review may still be made public.

Do you want your identity to be public for this peer review? For information about this choice, including consent withdrawal, please see our Privacy Policy.

**Reviewer #2: No**

***Response:*** Thanks for your important comments.

Special thanks to you for your good comments.

We tried our best to improve the manuscript and made some changes in the manuscript. According to the comments of reviewers, we have revised the manuscript and list specific changes here point by point. The changes have been highlighted in yellow in revised paper. The table line number of specific changes is based on the new revised manuscript.

We appreciate for Editors/Reviewers’ warm work earnestly, and hope that the correction will meet with approval.

Once again, thank you very much for your comments and suggestions.

***The list of specific changes***

1. **Page 2, line 16**

“small molecular weight” was removed.

2. **Page 7, line 12**

“and GAPDH was the internal reference” was removed.

3. **Page 8, line 2**

“; GAPDH, AB-P-R 001, GOODHERE BIOTECH, Hangzhou, China” was removed.

4. **Page 11, line 13-14**

“For western blotting, Image J was used to obtain the grayscale value of the target protein band which was further normalized with total protein.” was added.

5. **Page 14, line 10-11**

“GAPDH as the internal reference” was replaced by “normalization method using total protein existing on experimental blot”.

6. **Page 14, line 11-13**

“Results suggested that the expression of titin protein of 10kDa~18kDa, 18~23kDa, 23~30kDa, 30kDa, 42kDa, and 55 kDa significantly decreased in mutant mice heart tissue than in WT mice heart tissue (Figs 3B-3G)” was replaced by “Results showed a downward trend in the expression of titin proteins of 10kDa~18kDa, 18~23kDa, 23~30kDa, 30kDa, 42kDa, and 55 kDa in mutant mice heart tissue compared with WT mice heart tissue (Figs 3B-3G)”.

7. **Page 14, line 13**

“Additionally, the total TTN protein of KO mice was significantly lower than that of WT mice (S2 Fig).” was removed.

8. **Page 14, line 16-19**

“The expression of Titin of 10-18 kDa, 18-23 kDa, 23-30 kDa, 30 kDa exhibited an apparent decline in cardiac tissue of KO mice compared with WT mice. 42 kDa and 55 kDa titin of KO mice expressed a decreasing trend” was replaced by “There was no significant difference in the expression of Titin of 10-18 kDa, 18-23 kDa, 23-30 kDa, 30 kDa, 42 kDa and 55 kDa between KO mice and WT mice. But all these 6 titin isoforms expressed a downward trend in heart tissue of KO mice.”.

9. **Page 17, line 23-24**

“western blot results suggested a significant decrease in the protein expression level of some Titin isoforms caused by TTN-truncating variant c.13254T>G, especially low molecular weight Titin protein fragments” was replaced by “western blot results suggested a downward trend in the protein expression level of some Titin isoforms caused by TTN-truncating variant c.13254T>G”.

10. **Page 20, line 3**

“small molecular” was removed.

11. **Page 23, line 11-12**

“S2 Fig. (A) Quantification results of the total titin of KO mice and WT mice by WB. WB was carried out twice on KO mouse #2 and WT mouse L164 heart tissue samples. (B) Wilcoxon test showed that titin's total protein expression level was profoundly declined in KO mice than in WT mice.” was removed.
