# Supplementary material for: Integrated multi-omics approach revealed TTNtv c.13254T>G causing dilated cardiomyopathy in mice
Source: PLoS One. 2024 Oct 4;19(10):e0311670. doi: 10.1371/journal.pone.0311670 (PMC11452030; doi:10.1371/journal.pone.0311670)
Supplement: Attachment — Submitted filename: 01 Response to Reviewers.docx [file pone.0311670.s005.docx]

Dear editors and reviewers:

Thank you for your letter and for the reviewers’ comments concerning our manuscript entitled “*Integrated multi-omics approach revealed TTNtv c.13254T>G causing dilated cardiomyopathy in mice*” (PONE-D-23-40669). Those comments are all valuable and very helpful for revising and improving our paper, as well as the important guiding significance to our researches. We online submitted a rebuttal letter, a marked-up copy of the manuscript that highlights changes, an unmarked version of the revised paper, one re-prepared figure (Figure 3) processed by PACE, and an updated S1_raw_images. We have studied comments carefully and have made correction which we hope meet with approval. Revised portion are highlighted in yellow in the paper. The main corrections in the paper and the responds to the reviewer’s comments are as following:

**Responds and rebuttal to the comments of academic editor and reviewer(s):**

**Journal Requirements:**

1. Please review your reference list to ensure that it is complete and correct. If you have cited papers that have been retracted, please include the rationale for doing so in the manuscript text, or remove these references and replace them with relevant current references. Any changes to the reference list should be mentioned in the rebuttal letter that accompanies your revised manuscript. If you need to cite a retracted article, indicate the article’s retracted status in the References list and also include a citation and full reference for the retraction notice.

***Response:*** Thanks for your important comments. We have reviewed the reference list carefully. It is complete and correct without any retracted papers cited. No changes have been made to the reference list.

**Comments to the Author:**

1. If the authors have adequately addressed your comments raised in a previous round of review and you feel that this manuscript is now acceptable for publication, you may indicate that here to bypass the “Comments to the Author” section, enter your conflict of interest statement in the “Confidential to Editor” section, and submit your "Accept" recommendation.

Reviewer #2: (No Response)

Reviewer #3: All comments have been addressed

***Response:*** Thanks for your important comments. Thank you very much.

2. Is the manuscript technically sound, and do the data support the conclusions?

The manuscript must describe a technically sound piece of scientific research with data that supports the conclusions. Experiments must have been conducted rigorously, with appropriate controls, replication, and sample sizes. The conclusions must be drawn appropriately based on the data presented.

Reviewer #2: Partly

Reviewer #3: Yes

***Response:*** Thanks for your important comments. According to your suggestion, we’ve supplemented the gel image of the total protein used for normalization and deleted WT mice titin isoforms decreasing trend-related content from the article.

3. Has the statistical analysis been performed appropriately and rigorously?

Reviewer #2: Yes

Reviewer #3: Yes

***Response:*** Thanks for your important comments. Thank you very much.

4. Have the authors made all data underlying the findings in their manuscript fully available?

The PLOS Data policy requires authors to make all data underlying the findings described in their manuscript fully available without restriction, with rare exception (please refer to the Data Availability Statement in the manuscript PDF file). The data should be provided as part of the manuscript or its supporting information, or deposited to a public repository. For example, in addition to summary statistics, the data points behind means, medians and variance measures should be available. If there are restrictions on publicly sharing data—e.g. participant privacy or use of data from a third party—those must be specified.

Reviewer #2: Yes

Reviewer #3: Yes

***Response:*** Thanks for your important comments. Thank you very much.

5. Is the manuscript presented in an intelligible fashion and written in standard English?

PLOS ONE does not copyedit accepted manuscripts, so the language in submitted articles must be clear, correct, and unambiguous. Any typographical or grammatical errors should be corrected at revision, so please note any specific errors here.

Reviewer #2: Yes

Reviewer #3: Yes

***Response:*** Thanks for your important comments. Thank you very much.

**6. Review Comments to the Author:**

Please use the space provided to explain your answers to the questions above. You may also include additional comments for the author, including concerns about dual publication, research ethics, or publication ethics. (Please upload your review as an attachment if it exceeds 20,000 characters)

**Reviewer #2:** Thanks for suggested changes: However, there are minor concern and need to be addressed.

Major point:

(1) It would be better to submit complete gel picture of total protein (used for normalization) either under the main Western Gel in Figure or in Supplement.

***Response:*** Thanks for your important comments. The gel picture of the total protein used for normalization has been added to figure 3 as figure 3B, and to the supplementary file S1_raw_images. We first obtained gray values of titin isoforms and total protein using the software ImageJ. Then, titin isoforms were normalized by being divided by the gray value of the total protein. We further analyzed the difference in the normalized gray value of titin isoforms between KO mice and WT mice. The results showed no significant difference in the protein expression level of these titin isoforms between the two groups.

(2) Statistically, there is no differences in titin protein isomers between KO and WT. Moreover, there is negligible differences in the mean values of quantifications of all titin isomers between KO and WT. In that case, it not appropriate to describe them as "decreasing trend" in the text and figure legend. Critically, there is no changes. It is recommended to make changes and describe accordingly in results, discussion, and figure legend.

***Response:*** Thanks for your important comments. We’ve removed all the conclusions related to the decreasing trend of WT mice titin isoforms from the abstract, results, discussion and figure legend of the manuscript (Page 2, line 16; Page 14, line 10-13; Page 14, line 19; Page 17, line 22; Page 20, line 2).

Reviewer #3: The author has adequately addressed the comments of the reviewers from the previous revision. The statistics have been performed appropriately and rigorously, and the manuscript is presented in an intelligible fashion and written in standard English.

***Response:*** Thanks for your important comments.

**7.** PLOS authors have the option to publish the peer review history of their article (what does this mean?). If published, this will include your full peer review and any attached files.

If you choose “no”, your identity will remain anonymous but your review may still be made public.

Do you want your identity to be public for this peer review? For information about this choice, including consent withdrawal, please see our Privacy Policy.

Reviewer #2: No

Reviewer #3: No

*Response:* Thanks for your important comments.

Special thanks to you for your good comments.

We tried our best to improve the manuscript and made some changes in the manuscript. According to the comments of reviewers, we have revised the manuscript and list specific changes here point by point. The changes have been highlighted in yellow in revised paper. The table line number of specific changes is based on the new revised manuscript.

We appreciate for Editors/Reviewers’ warm work earnestly, and hope that the correction will meet with approval.

Once again, thank you very much for your comments and suggestions.

***The list of specific changes***

1. **Page 2, line 16**

The sentence “The TTNtv Y4370* caused a decrease in some isoforms of Titin protein.” was removed.

2. **Page 14, line 8**

“isoforms (Fig 3A)” was added.

3. **Page 14, line 10**

“(Fig 3B)” was added. “(Figs 3A)” was deleted.

4. **Page 14, line 10-13**

Sentence “Results showed a downward trend in the expression of titin proteins of 10kDa~18kDa, 18~23kDa, 23~30kDa, 30kDa, 42kDa, and 55 kDa in mutant mice heart tissue compared with WT mice heart tissue (Figs 3B-3G)” was replaced by “Results showed no significant differences in the expression of titin proteins of 10kDa~18kDa, 18~23kDa, 23~30kDa, 30kDa, 42kDa, and 55 kDa in mutant mice heart tissue compared with WT mice heart tissue (Figs 3C-3H)”.

5. **Page 14, line 15-16**

“(B) Western blot image of total titin protein of KO mice and WT mice.” was added.

6. **Page 14, line 16**

“(B)~(G)” was replaced by “(C)~(H)”.

7. **Page 14, line 19**

“But all these 6 titin isoforms expressed a downward trend in heart tissue of KO mice.” was removed.

8. **Page 17, line 22**

“In addition, western blot results suggested a downward trend in the protein expression level of some Titin isoforms caused by TTN-truncating variant c.13254T>G.” was deleted.

9. **Page 20, line 1**

“and” was added.

10. **Page 20, line 2**

“, and decreased expression level of titin isoforms” was removed.
